# Gastrin/CCK-B Receptor Signaling Promotes Cell Invasion and Metastasis by Upregulating MMP-2 and VEGF Expression in Gastric Cancer

**DOI:** 10.7150/jca.51854

**Published:** 2022-01-01

**Authors:** Yan Zhao, Qinrong Wang, Yi Zeng, Yuan Xie, Jianjiang Zhou

**Affiliations:** Key Laboratory of Endemic and Ethnic Diseases, Ministry of Education and Key Laboratory of Medical Molecular Biology, Guizhou Medical University, Guiyang, Guizhou, China.

**Keywords:** Gastrin, Cholecystokinin B receptor, Gastric cancer, MMP-2, VEGF

## Abstract

Accumulated evidence suggests that a functional loop composed of gastrin and cholecystokinin B receptor (CCK-BR) may exist in gastric carcinogenesis. However, this suggestion is not completely supported due to a lack of direct evidence, and the underlying mechanism is not completely understood. Here, we evaluated the effects of gastrin/CCK-BR signaling on the cell growth, invasion, and expression of MMP-2 and VEGF, as well as xenograft growth *in vivo*. Furthermore, we detected gastrin mRNA content in human gastric cancer tissues, metastatic lymph nodes, and adjacent nontumor tissues. We found that the forced gastrin could promote the proliferation, migration, and invasion of gastric cancer cells by upregulating the expression of MMP-2 and VEGF. Blocking gastrin/CCK-BR signal using either Proglumide, a CCK-BR antagonist, or shRNA against *GASTRIN* significantly inhibited the gastrin-promoting effects. *In vivo* study revealed that the tumor growth in nude mice inoculated with gastrin-overexpressed cells was significantly faster than control cells. The gastrin mRNA content in metastatic lymph nodes was higher in patients with gastric cancer than in primary gastric cancer and adjacent nontumor tissues. In conclusion, we provided direct evidence and possible mechanism of gastrin/CCK-BR signaling in the initiation and progression of gastric cancer.

## Introduction

Gastric cancer is one of the most prevalent cancers with high incidence and mortality, especially in Eastern Asia, including China, Japan, and Korea 1. Based on the current global cancer statistics from World Health Organization, the age-standardized rates (ASRs) of incidence and mortality of gastric cancer in the World, Eastern Asia, and China were 11.1, 22.4, and 20.6 (/10^5^) and 7.7, 14.6, and 15.9 (/10^5^), respectively, in 2020 2. Despite the decline in the prevalence of gastric cancer over recent decades, most cases are discovered at an advanced stage, and subsequently, most of the cancer patients die within a few years of diagnosis 3.

Gastrin, a peptide hormone of small molecule produced in G cells of the gastric antrum, is the central regulator of gastric acid secretion by promoting histamine secretion from enterochromaffin-like cells (ECL cells) and regulates the growth and differentiation of gastric mucosa 4. In pathological conditions like gastric cancer, gastrin exerts growth-promoting effects in the oxyntic mucosa and gastric epithelial cells 5. In the latest study by Sheng et al. 6, Cholecystokinin B receptor (CCK-BR), a seven-transmembrane G protein-coupled membrane receptor, was confirmed to be expressed mainly in ECL cells in the oxyntic mucosa of the stomach, and gastrin stimulated CCK-BR-positive progenitor cells to differentiate into gastric ECL cells. CCK-BR, by binding gastrin, produces gastrin/CCK-BR signaling in gastric ECL cells and mediates the physiological and pathological effects of gastrin, also known as gastrin receptor 7.

Whether gastrin has a dominant role in the development of gastric carcinoma has been under debate. Until recently, there was some accumulated evidence that gastrin is carcinogenic. In some patients with gastric carcinoma, serum gastrin levels are elevated, and gastrin and CCK-BR are co-expressed in some cancer tissues and cancer cell lines 8. The exogenous gastrin 17 has also been shown to play an essential role in stimulating the growth, migration, and invasion of several gastrointestinal cancer cell lines *in vitro*, including gastric cancer cell lines 9. These findings implied that a functional autocrine/paracrine loop composed of gastrin and CCK-BR might exist in gastric carcinogenesis. However, this theory is not completely supported due to a lack of direct evidence, and further research is needed.

Our previous study observed that the CCK-BR and gastrin mRNA and protein were co-expressed in human gastric cancer cell lines and gastric cancer tissues, and downregulation of CCK-BR affected cell proliferation and apoptosis in gastric cancer cells 10. In this study, we further investigated the effects of gastrin/CCK-BR autocrine/paracrine signaling on cell invasion and metastasis and underlying mechanisms in gastric cancer. Our findings will provide more evidence that the gastrin/CCK-BR axis may serve as a potential molecular target for the treatment of this malignant disease. To our knowledge, this is the most comprehensive study of its kind to date, and it is also the first paper to observe that gastrin could upregulate the expression of MMP-2 and VEGF.

## Materials and Methods

### Tissue samples

Tissue samples, including gastric cancer and adjacent nontumor tissues and metastatic lymph nodes, were collected from 50 gastric cancer patients during surgery at the Affiliated Hospital of Guizhou Medical University, China, between January 2014 and June 2015, and half of the tissue was used for pathological analysis. These samples were immediately embedded in RNAlater and stored at -80°C. The diagnoses were confirmed by two pathologists. Of the 50 patients, 38 were male, and 12 were female. Their median age was 68 (range 38-82) years. Thirty-six (72%) cancers were adenocarcinoma of the intestinal type, and fourteen (28%) were adenocarcinoma of the diffuse type. Thirty-five (70%) cancers occurred in the gastric antrum, and fifteen (30%) were in the gastric corpus. All subjects gave their informed consent for inclusion before they participated in the study. The study was conducted according to the Declaration of Helsinki, and the protocol was approved by the Ethics Committee of Guiyang Medical University (No: 2014(41), *March 7*, 2014).

### Cell culture, vector construction, and stable cell line generation

Human gastric carcinoma cell line AGS (catalog no. CRL-1739TM) and SGC-7901 cells were purchased from the American Type Culture Collection (USA) and the Cell Bank of Type Culture Collection of the Chinese Academy of Sciences (China), respectively. AGS cells were isolated from a biopsy specimen of an untreated human adenocarcinoma of the stomach 11, and SGC-7901 cells were isolated from a metastatic lymph node of a Chinese patient with gastric adenocarcinoma 12. The morphology of the two cell lines is epithelial, and they originated, respectively, from malignant and metastatic epithelial cells and not from ECL cells of the stomach. Cells were cultured in Roswell Park Memorial Institute (RPMI) 1640 medium supplemented with 10% heat-inactivated fetal bovine serum (Gibco), 100 U/ml penicillin, and 100 μg/ml streptomycin (Gibco) at 37°C in a humidified incubator with 5% CO2.

Total RNA was extracted from tissue samples and reverse-transcribed to cDNA. Gastrin gene was amplified by polymerase chain reaction (PCR). The primer sequences are (5′-3′): GCTCTAGACCATGCAGCGACTATGTGTGTAT and GCCCTAGG TCAGTTTTTCAGGGGACAG. The PCR products were ligated to a pcDNA3.1 plasmid to construct the pcDNA/*gastrin* vector. The vectors were identified by sequencing. Cells were transfected with the pcDNA/gastrin or pcDNA3.1 vector using LipofectAMINE 2000 (Invitrogen). Plates were incubated for 2 weeks allowing cells to form colonies using a concentration of 0.3 mg/mL G418 (Sigma Aldrich) for AGS cells and 0.15 mg/mL G418 for SGC-7901 cells. The cell lines stably expressing gastrin were established and named AGS/Gastrin and SGC-7901/Gastrin. The corresponding controls were named AGS/pcDNA and SGC-7901/pcDNA.

Small interfering RNA (siRNA) targeting the gastrin transcript was designed. Sequences were (5′-3′): UCCAUCCAUAGGCUUCUUCUU. The siRNA and scrambled siRNA were ligated to a Psilencer3.1 plasmid to construct Psilencer3.1/Gas-siRNA and Psilencer3.1/ scrambled, which were identified by sequencing. The vectors were used to transfect the AGS cells using x-tremeGENE HP DNA Transfection Reagent (Roche). The clonal selection was conducted by culturing with G418 (0.4 mg/mL) followed by serial dilution of the cells. Stable transfection clones with low expression of *GASTRIN* gene (AGS/Gas-siRNA) and control clone (AGS/scrambled) were identified by quantitative PCR (qPCR) and ELISA analysis.

### Proglumide treatment

Proglumide, a CCK-BR antagonist, was used to treat gastrin-overexpressing cells at a final concentration of 5 mmol/L.

### Immunocytochemistry

Cells were cultured on coverslips in 6-well plates for 24 h, and the coverslips were retained in absolute alcohol for 30 min. Then, cells were incubated with 0.3% Triton X-100 for 20 min and blocked in 3% hydrogen peroxide (H_2_O_2_) for 30 min at room temperature. Rabbit anti-gastrin antibody (Abcam; ab53085; 1:100), mouse anti-chromogranin A antibody (Beijing Zhongshan Golden Bridge Biotech, China; TA506095; 1:100), and morse anti-synaptophysin antibodies (Sigma; S-5768; 1:250) were used to detect gastrin, chromogranin A, and synaptophysin expression, respectively. The EnVision™ universal antibody and DAB (Dako) were used to visualize the primary antibodies following the manufacturer's instructions. Coverslips were counterstained with hematoxylin (Sigma). Images were collected using a microscope (Olympus).

For immunofluorescence, a fluorescent secondary antibody was incubated with the coverslips in a humid box at room temperature for one hour. Cell nuclei were counterstained with DAPI (Sigma-Aldrich, MO, USA). Images were acquired on a confocal microscope (Olympus, Japan).

### Quantitative RT-PCR analysis

Total RNA was extracted from 50 mg of tissues or 1×10^6^ cells with TRIzol (Invitrogen), treated with DNase I (RNase-free), and then reverse-transcribed to cDNA. Quantitative RT-PCR (RT-qPCR) was used to quantify gastrin mRNA levels using the TaqMan gene expression assay from Applied Biosystems (gastrin probe: Assay ID Hs00174945; β-actin probe: 4333762T). The mRNA levels of matrix metalloproteinase 2 (MMP-2) and vascular endothelial growth factor (VEGF) were quantitated by RT-qPCR with SYBR Green (Applied Biosystems). The primer sequences are as follows (5′-3′): MMP-2: AGGGCACATCCTCTGACAGC and CGGTCGTAGTCCTCAGTGGT. VEGF: AGCCTTGCCTTGCTGCTCTAC and TGATGATTCTGCCCTCCTCCTT. β-actin: TGGAGAAAATCTGGCACCAC and GAGGCGTACAGGGATAGCAC. β-actin was used as a normalization control, and the relative mRNA levels of the target genes were analyzed by a comparative Ct method (2^-ΔΔCt^). Each sample was assayed in triplicate in three independent experiments, and the results are expressed as the mean ± SD.

### Western blot analysis

Whole-cell extracts were prepared by resuspending cell pellets in a RIPA buffer supplemented with a cocktail of protease (Roche). The protein concentrations were quantified by the Bradford method. A total of 30 μg of protein extract was subjected to 10% SDS-PAGE gel electrophoresis, transferred to a polyvinylidene difluoride (PVDF) membrane (Millipore), and blotted overnight with rabbit polyclonal anti-MMP-2 antibody (sc-10736, 1:800, Santa Cruz), rabbit polyclonal anti-VEGF antibody (sc-507, 1:350, Santa Cruz) and mouse monoclonal anti-glyceraldehyde-3-phosphate-dehydrogenase (GAPDH) antibody (sc-365062, 1:8000, Santa Cruz) in 5% BSA in Tris-buffered saline and 0.01% Tween-20. Peroxidase-conjugated secondary antibodies (sc-2371, 1:5000, Santa Cruz) were used and developed with the chemiluminescence reagent ECL Plus using hyperfilm (Amersham Biosciences). Quantification of the western blots was performed using Quantity One software. Each experiment was conducted three times, and a representative result was shown.

### ELISA

According to the manufacturer's instructions, Gastrin, MMP-2, and VEGF concentrations in medium from cultured cells were detected using ELISA kits (R&D Systems).

### MTT assay

Cells were cultured in a 96-well plate for 48 h, and 3-(4,5-dimethyl-2-thiazolyl) -2,5-diphenyl-2-H-tetrazolium bromide (MTT) solution (Sigma) was added to the wells. After 4 h of incubation, DMSO was added to each well. The absorbance at 570 nm was measured, and the cell growth rate was calculated according to the formula: Cell growth rate = (*A_570_* in experimental group/*A_570_* in control group) ×100%.

### Colony formation assay

First, 1.2 % low melting agarose was added to a 6-well plate to pave the base agar layer, and cells were mixed with 0.7 % low melting agarose to pave the cell agar layer on the base layer. Then, cells were incubated for 2 to 3 weeks until the formation of cell colonies. Colonies were counted from 10 fields under a microscope, and the average colony numbers and colony formation rate were calculated following the formula: Colony formation rate = (colonies in experimental group /colonies in control group) ×100%.

### Cell wound healing assay

Cells were incubated for 24 to 48 h until a monolayer in a 12-well plate and a “wound field” was generated across the well center. Cells were cultured for migration into the wound field from either side of the gap. Twenty-four hours later, images were taken under an inverted microscope at 100× magnification. The relative migration distance was defined as the difference in the distance between both wound edges at 0 h and after 24 h culture.

### Cell migration and invasion assays

Cell migration and invasion ability were assessed using Corning Transwell® polycarbonate membrane cell culture inserts without and with Matrigel coating (BD Biosciences). Cells (2 x 10^4^) were harvested and plated onto the upper chamber of the Transwell with a culture medium supplemented with 0.1% FBS. The chambers were incubated for 24 h with a culture medium supplemented with 30% FBS added in the lower chamber of the Transwell. Non-moved cells in the upper chamber were removed, and chambers were stained with crystal violet. The stained cells were counted from ten fields under a microscope at 400 × magnification, and the average migration and invasion cell numbers were calculated. All experiments were repeated three times.

### * In vivo* studies

Twelve specific pathogen-free (SPF) normal female BALB/c nude mice with bodyweight from 13 to 16 g and aged 2 to 3 weeks were purchased from the Experimental Animal Center of Shanghai XIPUER-BIKAI laboratory (animal certification number: SCXK2007-0007) and housed in a specific pathogen-free environment in the Laboratory Animal Center of Guizhou Medical University.

Next, 5×10^6^ cells were injected subcutaneously in the right lateral axilla of 5-week-old female BALB/c nude mice (n = 6 per group). Tumor growth was monitored over time for 5, 10, 15, 20- and 25-days using calipers, and tumor volume was calculated using the following formula: Tumor volume = (lengh×width^2^)/2. Twenty-five days later, these mice were sacrificed by cervical dislocation, and tumors were removed and weighed. The data are the average of six mice per group. All experiments on animals were approved by the Committee on Animal Care of Guizhou Medical University (No: 1401035, *March 6*, 2014). All animals received humane care according to the criteria outlined in the “Guide for the Care and Use of Laboratory Animals” by the National Research Council. The authors ensure that the experimental design, analysis, and reporting followed the ARRIVE guidelines (https://www.nc3rs.org.uk/arrive-guidelines).

### Statistical analysis

Results are expressed as means ± SD. Statistical analyses were performed using SPSS 15.0 software. One-way analysis of variance (ANOVA) and Student's t-test were used to analyze the data. *P*<0.05 (two-sided) was considered statistically significant.

## Results

### Gastrin/CCK-BR signaling promotes the growth of gastric cancer cells

**Figure 1:**
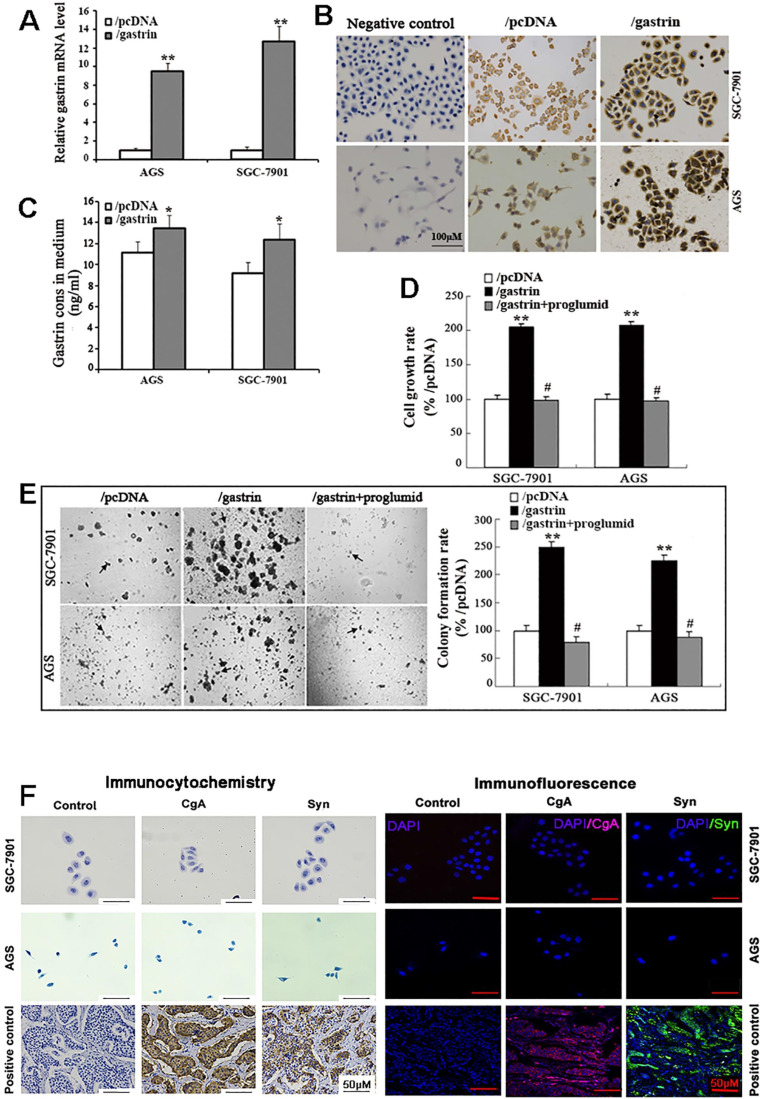
** Identification of the stable cell lines overexpressing gastrin and detection of cell growth.** (**A-C**) Identification of stable cell lines. AGS and SGC-7901 cells were transfected with pcDNA/*gastrin* and pcDNA3.1 vectors to generate stable cell lines by G418 screening. The gastrin mRNA level (**A**), protein expression (**B**), and concentrations in the medium (**C**) of stable cell lines were detected by RT-qPCR, immunocytochemistry, and ELISA, respectively. Gastrin mRNA level was normalized to β-actin, and AGS/pcDNA and SGC-7901/pcDNA were used as controls. Results are shown as relative to the value of 1 assigned to controls (**A**). Gastrin immunocytochemistry staining was performed, in which that the antibody against gastrin was replaced by PBS was used as a negative control (**B**). (**D**) Detection of cell growth rate by MTT. Stably transfected cells were cultured for 48 h with or without proglumide (5 mmol/L) in sextuplicate, and then each well was supplemented with 20 µL of MTT solution (5 mg/mL). After 4 h of incubation, 200 µL of DMSO was added to each well, and the absorbance at 570 nm was measured. The cell growth rate was calculated according to the formula: Cell growth rate = (*A*_570_ in experimental group/*A*_570_ in control group) ×100%. (**E**) Detection of colony formation in soft agar. Colonies were counted from 10 fields, and average colony numbers were calculated. The colony formation rate was calculated following the formula: Colony-forming rate = (colonies in experimental group/colonies in control group) ×100%. All data are expressed as the mean ± SD. The images were representative of three independent experiments. ^*^P < 0.05 and ^**^P < 0.01 vs. /pcDNA group; ^#^*P* < 0.05 vs. /gastrin group. (**F**) Immunocytochemistry and immunofluorescence staining for chromogranin A (CgA) and synaptophysin (Syn) in SGC-7901 and AGS cells. PBS instead of the primary antibody was used as a control. The gastric neuroendocrine tumor was used as a positive control.

The full-length gastrin sequences acquired from normal gastric antrum mucosa were successfully inserted into the pcDNA3.1 vector, which was confirmed by sequencing. The pcDNA3.1/gastrin vector was used to transfect the gastric cancer AGS and SGC-7901 cells to establish stable gastrin-overexpressing cell lines. We found that the expression of gastrin mRNA and protein and gastrin concentrations in the medium in the cell lines stably overexpressing gastrin were higher than those in empty vector-transfected cells, suggesting that the stable cell lines overexpressing gastrin and control cell lines were successfully acquired, named AGS/gastrin, SGC-7901/gastrin, AGS/pcDNA, and SGC-7901/pcDNA, respectively (Figure 1A-C). In gastrin-overexpressed stable cells, the cell growth rate and colony formation rate in soft agar were increased by 105.22% and 149.47% for SGC-7901 and 107.43% and 124.79% for AGS, respectively, compared with the corresponding control cells (Figure 1D, E).

Proglumide, a CCK-BR antagonist, can bind to CCK-BR with gastrin competition to block the gastrin/CCK-BR system. After the stable cells overexpressing gastrin were exposed to proglumide (5 mmol/L) for 48 h, both cell growth rate and colony formation rate were significantly decreased (Figure 1D, E), suggesting that the gastrin/CCK-BR loop is involved in gastric cancer cell growth.

Like gastric cancer SGC-7901 and AGS cells, ECL cell is also epithelial in origin. Therefore, we examined the two gastric cancer cells for ECL markers chromogranin A (CgA) and synaptophysin (Syn) by immunocytochemistry and immunofluorescence, and negative staining was observed for CgA and Syn in both SGC-7901 and AGS cells (Figure 1F). The result further confirmed that SGC-7901 and AGS cell lines did not originate from gastric ECL cells.

### Gastrin/CCK-BR signaling promotes the migration and invasion of gastric cancer cells

**Figure 2:**
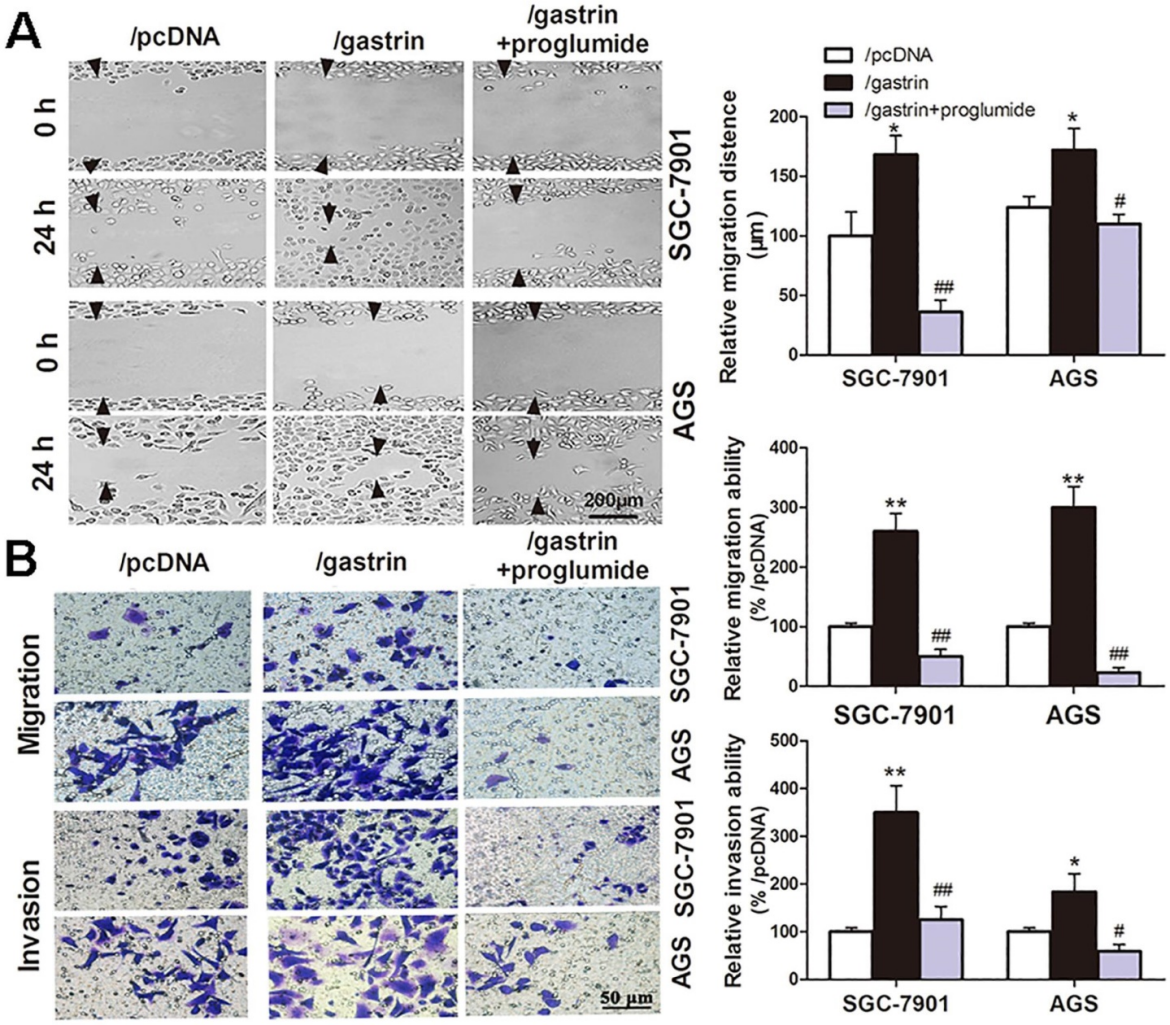
** Gastrin promotes the migration and invasion of gastric cancer cells.** (**A**) Wound healing assays for evaluating the cell migration of stable cell lines. Images were taken at 0 h and 24 h culture under an inverted microscope at 100× magnification. The images shown are representative of three independent experiments. Relative migration distance was defined as the difference in the distance in the wound edge (arrow) at 0 h and 24 h culture. (**B**) Transwell assays for assessing the migration and invasion ability of stable cells. Cells were seeded in the upper chamber of the Transwell, which was coated with (invasion) or without (migration) Matrigel. After incubation for 24 h, the cells in the upper chambers were removed. The cells invading or migrating to the lower chamber were stained by crystal violet and counted in ten fields under a microscope at 400 × magnification. The average migration and invasion cell numbers were calculated. The images were taken under an inverted microscope at 100× magnification. The results shown are representative of three independent experiments. ^*^P < 0.05 and ^**^P < 0.01 vs. /pcDNA group; ^#^*P* < 0.05 and ^##^*P* < 0.01 vs. /gastrin group.

Many experiments have shown the proliferation-promoting effects of gastrin in gastric cancer cells. However, whether the gastrin/CCK-BR axis is crucial for the invasion and metastasis of gastric cancer cells remains unclear. Through cell wound healing and Transwell assays, we discovered that overexpressing gastrin not only promoted the cells to migrate into the wound area but also promoted the cells in the upper chamber of the Transwell to migrate across the membrane or invade across Matrigel into the lower chamber. After using proglumide to interfere with the gastrin/CCK-BR axis, the promoting effects of gastrin on cell migration and invasion were significantly suppressed (Figure 2).

### Silencing gastrin inhibits the proliferation, migration, and invasion of gastric cancer cells

**Figure 3:**
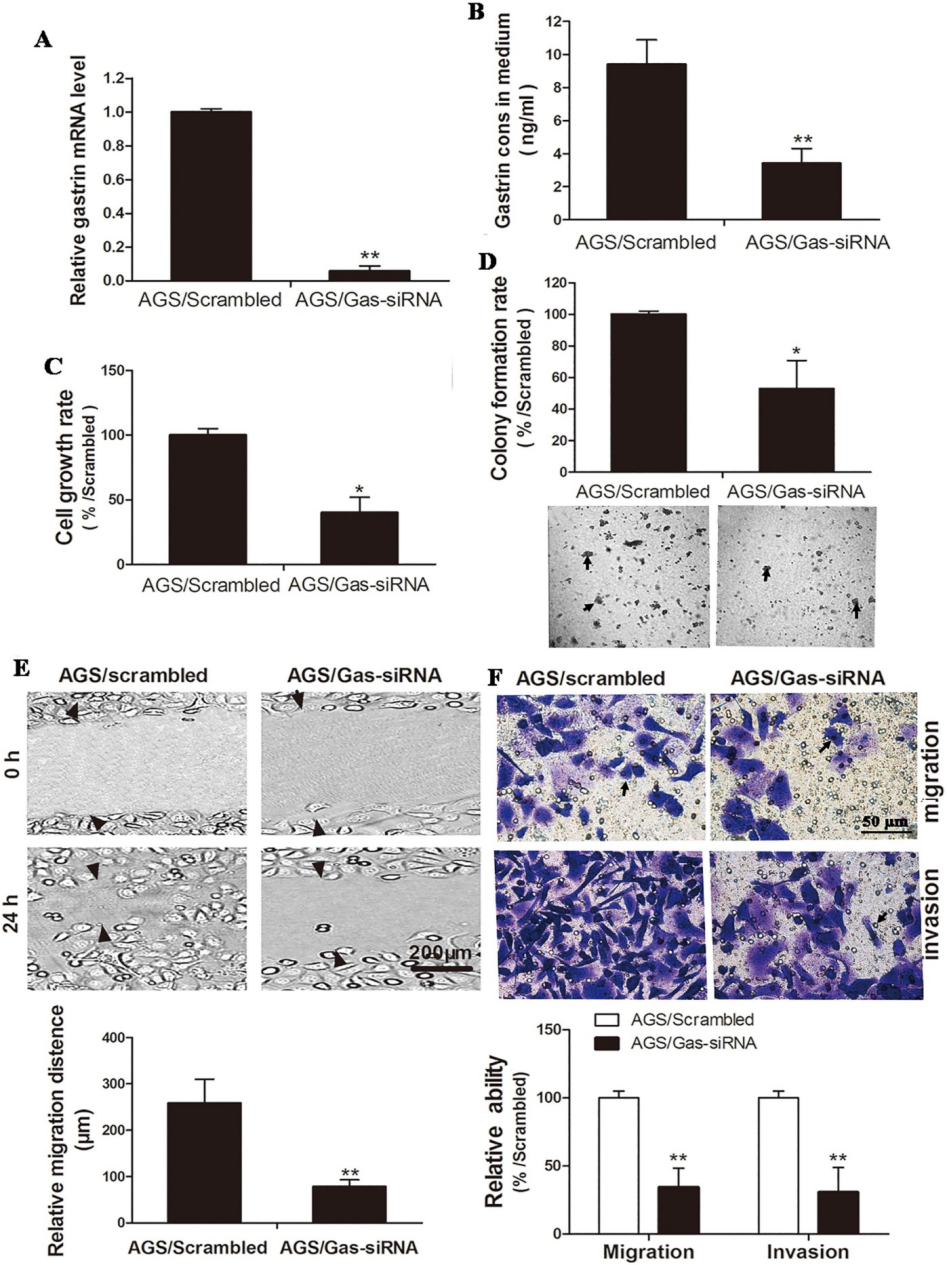
** Silencing gastrin inhibits the growth, migration, and invasion of gastric cancer cells.** (**A** and **B**) Identification of stable AGS cells knocking down gastrin**.** AGS cells were transfected with the pSilencer3.1/Gas-siRNA and pSilencer3.1/scrambled vectors to establish stable cells by G418 screening, named AGS/Gas-siRNA and AGS/scrambled, respectively. The gastrin mRNA level (**A**) and concentration in the medium (**B**) of stable AGS cells were detected by RT-qPCR and ELISA, respectively. Gastrin mRNA level was normalized by β-actin, and AGS/scrambled was used as a control. Results are shown as relative to a value of 1 assigned to the control. (**C** and **D**) Silencing gastrin inhibits the cell growth and colony formation efficiency in stable cell lines using MTT assay (**C**) and soft-agar colony-forming assay (**D**). (**E** and **F**) Silencing gastrin suppresses the migration and invasion ability of gastric cancer cells by wound healing (**E**) and Transwell (**F**) assays. The experimental methods are described in Figure 1 and Figure 2, respectively. All data are expressed as the mean ± SD. The images are representative of three independent experiments.^ *^P < 0.05 and ^**^P < 0.01 vs. AGS/scrambled group.

Gastrin-specific shRNA and scrambled shRNA were ligated to a pSilencer3.1 vector to construct the pSilencer3.1/Gas-siRNA and pSilencer3.1/scrambled vectors. Both vectors were transfected into AGS cells to generate the cell line with stable knockdown of gastrin and control cell line. In contrast to the results described above that overexpression of gastrin promoted the growth and invasion of gastric cancer cells, the growth, migration, and invasion of the gastrin knockdown cells were significantly inhibited (Figure 3).

### Gastrin promotes tumor formation in nude mice and lymph node metastasis in patients with gastric cancer

**Figure 4:**
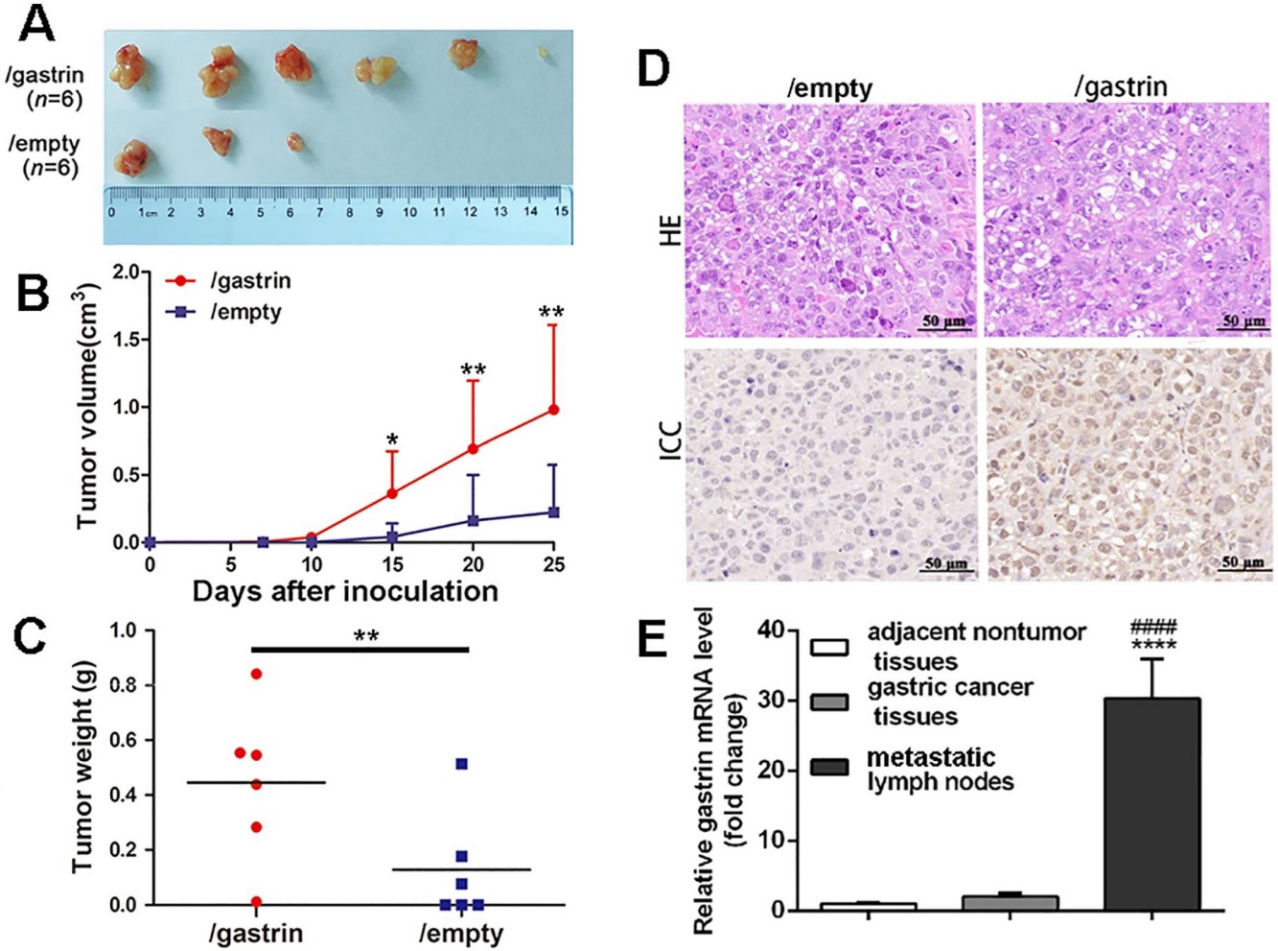
** Gastrin promotes tumor growth and metastasis *in vivo*.** (**A-C**) BALB/c nude mice were subcutaneously inoculated with SGC-7901/gastrin and SGC-7901/empty cells in the right lateral axilla at 5×10^6^ cells (*n* = 6 per group). Tumor dimensions were measured every 5 days using a caliper, and volume was calculated following the formula: Tumor volume = (length × width^2^)/2. Twenty-five days later, tumors were surgically removed from mice and weighed. All data are expressed as the mean ± SD derived from six mice per group. ^*^P < 0.05 and ^**^P < 0.01vs. /empty group. (**D**) HE and immunohistochemistry staining of gastrin were performed on the paraffin-embedded tumor tissues from nude mice following standard procedure. The images were representative of three independent experiments. (**E**) Gastrin mRNA levels in gastric cancer tissues, metastatic lymph nodes, and adjacent nontumor tissues were measured by RT-qPCR from RNA extracted from surgical resection from 50 patients with gastric cancer. Beta-actin was used as an internal control. Results are shown relative to a value of 1 assigned to adjacent nontumor tissues after normalization to the internal β-actin mRNA level. ^****^P< 0.0001 vs. adjacent nontumor tissues; ^####^*P*<0.0001 vs. gastric cancer tissues.

To further explore the effects of gastrin *in vivo*, the gastrin-overexpressed SGC-7901 cells and control cells were transplanted subcutaneously into BALB/c nude mice. The results indicated that the tumor formation rate in nude mice from gastrin-overexpressed cells (100%) was higher than that of control cells (50%) (Figure 4A). Fifteen days after inoculation, the volume of transplanted tumors in two groups began to appear different. Twenty-five days later, the volume and weight of tumors surgically removed from mice were significantly increased in the gastrin-overexpressed group compared to the control group (Figure 4B-C), and gastrin was highly expressed in tumor tissues of nude mice in the gastrin-overexpressed group (Figure 4D). Quantitative detection also found that gastrin mRNA levels in metastatic lymph nodes were 11 times higher than that in primary gastric cancer and 30 times that in adjacent nontumor tissues in patients with gastric cancer. However, there was no significant difference in the gastrin mRNA content between the other two groups (Figure 4E).

### Gastrin/CCK-BR signaling promotes the expression and secretion of MMP-2 and VEGF in gastric cancer cells

**Figure 5:**
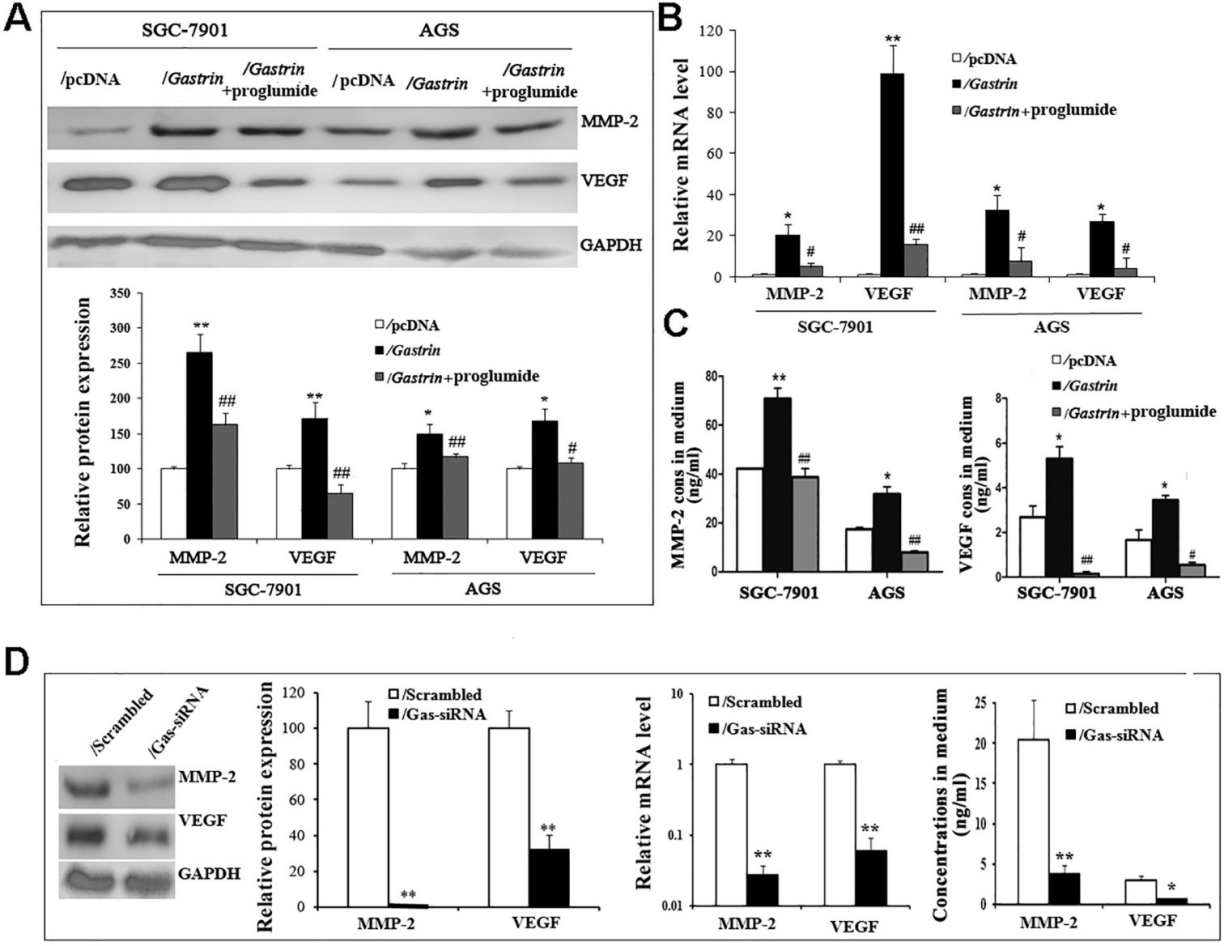
** Gastrin promotes the expression and secretion of MMP-2 and VEGF.** (**A**) The protein levels of MMP-2 and VEGF were determined in stable cell lines by western blot. GAPDH expression was used as an endogenous reference. (**B**) The mRNA levels of MMP-2 and VEGF were quantified in stable cell lines by RT-qPCR. MMP-2 and VEGF expression were normalized by β-actin, and AGS/pcDNA and SGC-7901/pcDNA were used as controls, respectively. Results are shown relative to a value of 1 assigned to controls. (C) Concentrations of MMP-2 and VEGF in the medium of stable cell lines were detected by ELISA. Data are expressed as the mean ± SD from three independent experiments (^*^P < 0.05 and ^**^P < 0.01 vs. / pcDNA; ^#^*P* < 0.05 and^ ##^*P* < 0.01 vs. /gastrin). (**D**) Protein and mRNA levels and concentrations in the medium of MMP-2 and VEGF were measured in gastrin-knocked down AGS cells by western blot, RT-qPCR, and ELISA, respectively. MMP-2 and VEGF expression was normalized by GAPDH, and AGS/scrambled was used as an internal control. Results are shown relative to a value of 1 assigned to controls. Data are expressed as the mean ± SD from three independent experiments (^*^P < 0.05 and ^**^P < 0.01 vs. / pcDNA).

MMP-2 and VEGF have been well-characterized by promoting cancer cell invasion and metastasis via degrading the extracellular matrix and increasing tumor angiogenesis. To determine whether the pro-invasive and -metastatic effects of gastrin involve MMP-2 and VEGF in gastric cancer cells, we detected the mRNA and protein expression and secretion of MMP-2 and VEGF in gastrin-overexpressed and gastrin-knocked down cells. The findings revealed that compared to the control groups, the levels of mRNA and protein and concentrations in medium of both MMP-2 and VEGF were significantly increased in gastrin-overexpressed cells, after silencing gastrin and proglumide treatment, the promoting effects were significantly inhibited (Figure 5).

## Discussion

Under physiologic conditions, gastric acid was secreted from parietal cells to aid in the digestion of food, absorption of minerals, and killing of food-derived bacteria. Multiple pathways are involved in the regulation of gastric acid secretion, including the neuronal pathways mediated by the enteric nervous system (ENS) and endocrine pathways mediated by the enteroendocrine cells in the gastrointestinal mucosa, the latter involving gastrin, histamine, acetylcholine, somatostatin, etc. 13. Among them, gastrin secreted from antral G cells, histamine released from oxyntic ECL cells, and acetylcholine released from antral and oxyntic intramural neurons (vagus and enteric neurons) are three main stimulants of acid secretion, while somatostatin, released from oxyntic and antral D cells, is the primary physiological inhibitor of acid secretion 14. In parallel, gastric parietal cells express specific receptors for these hormones, such as CCK-BR for gastrin, H2 receptor for histamine, muscarinic receptor (M3) for acetylcholine, and SST2 receptor for somatostatin. More importantly, gastric ECL cells also express CCK-BR, M3, and SST2 receptors 15. In these hormones, gastrin plays an important regulatory role in gastric acid secretion via binding to the CCK-BR, present on the major ECL cells and minor gastric parietal cells. Although CCK-BR is present on parietal cells, the direct effect of gastrin on acid secretion in humans is likely minor. Gastrin stimulates acid secretion in humans mainly by binding to CCK- BR on gastric ECL cells to stimulate ECL cells to secrete histamine. Histamine, in turn, diffuses to and binds to histamine H2 receptors on parietal cells to stimulate these cells to secrete hydrochloric acid through the activation of adenylate cyclase 16,17. In addition, Vagal efferent mediated by the ENS stimulates G cell to secrete gastrin through gastrin-releasing peptide (GRP), and ECL cells and parietal cells to release histamine and hydrochloric acid, respectively, through binding of acetylcholine to the M3 receptor on the two cells 16. As the main inhibitor of acid secretion, somatostatin inhibits parietal cell secretion directly and indirectly by inhibiting histamine secretion from ECL cells and gastrin secretion from G cells through binding to the SST2 receptor. The cholinergic signal can also accelerate acid secretion by inhibiting somatostatin release from D cells 18.

Taken together, the vagus nerve and numerous cell types in gastric mucosa secrete the neurotransmitters and hormones to jointly regulate the homeostasis of gastric acid secretion in the resting and food-stimulating parietal cells, which prevents damage to the gastric mucosa. However, in recent years, studies have found that gastrin is not only a secretagogue but a trophic hormone that promotes the growth and renewal of the gastric epithelium 19,20.

Growing evidence shows that in addition to the expression of gastrin in the endocrine G cells of the stomach, *GASTRIN* and *CCK-BR* genes also become overexpressed in nonendocrine gastric cancer epithelial cells 21. Our study and other data confirmed the co-expression of gastrin and CCK-BR in several cancers, including gastric adenocarcinoma, hepatocellular carcinoma, lung squamous cell carcinoma, and infiltrating ductal carcinoma of the breast, and cancer cell lines by cancer tissue microarray, immunohistochemistry, and immunocytochemistry 22, 23. These data support the notion that gastric cancer cells may express their intrinsic gastrin to produce a growth-promoting signal using an autocrine/paracrine mechanism.

In human beings, the amidated gastrin-17 is the central regulator in the maintenance and organization of the gastric mucosa and plays a pivotal role in gastrin-mediated physiological and pathological processes 24. Therefore, commercially available gastrin 17 (defined as G17) was used to treat gastric cancer cell lines *in vitro* to assess the effects of gastrin. Rao and Kun's groups reported that G17 treatment stimulated the proliferation and migration of gastric cancer cells via activating autophagy 8, 25. G17 was also observed to induce the migration and invasion of gastric cancer SGC-7901 cells through the β-catenin/TCF-4 pathway 26. Bhandari and Kim found that gastrokine 1 and connective tissue growth factor (CTGF) inhibited G17-induced cell proliferation, migration, and invasion of gastric cancer cells 27, 28. However, these findings are limited because G17, as exogenous gastrin, cannot be used to fully and effectively evaluate the effects of gastrin secreted by gastric cancer cells in the body.

In this study, we established six gastric cancer cell lines with enhancement or inhibition of gastrin/CCK-BR signaling by overexpression or knockdown of gastrin in cells to confirm that a functional gastrin/CCK-BR loop existed in gastric cancer cells. Gastrin, released into the medium by cancer cells and binding to CCK-BR on cancer cells, stimulated the proliferation, migration, and invasion of gastric cancer cells *in vitro*. Using proglumide, a CCK-BR antagonist, to block this signaling axis could inhibit the growth-promoting role of gastrin. Moreover, we observed that gastrin overexpression significantly promoted tumor formation in nude mice. More importantly, our findings did not show differential mRNA expression of gastrin between cancer and adjacent nontumor tissues of the patients with gastric cancer, even though the metastatic lymph nodes of these patients exhibited significantly higher expression of gastrin mRNA compared with the other two groups. This result was not completely consistent with that of cell line studies *in vitro*. It was also inconsistent with the report by Goetze, who found that gastrin mRNA was detectable in all of 20 gastric carcinomas tissues by RT-qPCR 29. However, in Goetze's report, gastrin mRNA levels were not compared with the surrounding tissues free of tumor cells and metastatic lymph nodes.

In earlier research using murine models, high and low expression of gastrin were closely associated with an increased risk of gastric cancer. Wang and others reported that insulin-gastrin (INS-GAS) transgenic mice with overexpression of gastrin in the pancreatic b-cells developed invasive gastric corpus cancer at 20 months of age, and infecting these mice with *Helicobacter pylori* (*H. pylori*) accelerated the development of gastric cancer 5, 30. However, the gastrin-knocked out (GAS-KO) transgenic mice were also observed to develop gastric antral cancer due to hypochlorhydria and bacterial overgrowth induced by the knockout of gastrin 31. The pathological analysis confirmed that the histologic changes observed in GAS-KO mice were similar to the precursor lesions progressing to gastric cancer, particularly atrophic gastritis, in human subjects. Atrophic gastritis was often present in the surrounding mucosa adjacent to gastric cancer 32. Our finding in human subjects was similar to GAS-KO mice. We speculate that a gastric microenvironment composed of *H. pylori*, atrophic gastritis, and inflammation, which are commonly present in the gastric mucosa adjacent to cancer, may inhibit the local expression of gastrin by cancer cells. With the metastasis of neoplasm cells to lymph nodes, the inhibitory action is abolished, and gastrin is highly expressed again in lymph nodes. However, this finding needs to be further confirmed.

Matrix metalloproteinases are a family of extracellular endopeptidases capable of degrading all extracellular matrix components, in which MMP-1 and MMP-2 are the two major components of the family and are believed to be mainly associated with tumor invasion and metastasis 33. VEGF is the most potent and specific tumor angiogenesis promoter, which is a fundamental process in tumor growth and metastasis 34. It was confirmed that the MMP-2 and VEGF were highly expressed in gastric cancer tissues and cells, and the overexpression of MMP-2 and VEGF was closely linked to the invasiveness and metastatic progress of cancer cells 35. VEGF was released excessively from gastric cancer cells into the serum to exert its pro-angiogenic effects 36. However, the mechanism for the overexpression of MMP and VEGF in gastric cancer tissues and cells is poorly understood. In this study, we found that high gastrin expression promoted MMP-2 and VEGF expression and secretion, and blocking gastrin/CCK-BR signaling inhibited the promoting effects, suggesting that gastrin/CCK-BR signaling facilitated the invasion and metastasis by promoting MMP-2 and VEGF expression in gastric cancer. Although the main effect of VEGF is by stimulation of blood supply which cannot play any role in cell cultures, our results confirm that gastrin/CCK-BR signaling could promote VEGF expression and release in gastric cancer cells, which may be a significant contributing factor for high tissue expression and release of VEGF in gastric cancer.

In conclusion, the present findings revealed a gastrin/CCK-BR autocrine/paracrine signaling loop in gastric cancer cells, which plays a significant role in the invasion and metastasis of cancer cells by promoting MMP-2 and VEGF expression. The data will provide more evidence that the gastrin/CCK-BR axis may serve as a potential molecular target for the treatment of this malignant disease.

## Abbreviations

CCK-BR: Cholecystokinin B receptor
MMP-2: Matrix metalloproteinase 2
VEGF: Vascular endothelial growth factor
ASRs: Age-standardized rates
PVD: Polyvinylidene difluoride
GAPDH: Glyceraldehyde-3-phosphate-dehydrogenase
MTT: 3-(4,5-dimethyl-2-thiazolyl)-2,5-diphenyl-2-H-tetrazolium bromide
